# Estimation of Articular Cartilage Surface Roughness Using Gray-Level Co-Occurrence Matrix of Laser Speckle Image

**DOI:** 10.3390/ma10070714

**Published:** 2017-06-28

**Authors:** Doaa Youssef, Hatem El-Ghandoor, Hamed Kandel, Jala El-Azab, Salah Hassab-Elnaby

**Affiliations:** 1 Department of Engineering Applications of Laser, National Institute of Laser Enhanced Sciences (NILES), Cairo University, Giza Governorate 12613, Egypt; jala@niles.edu.eg (J.E.-A.); selnaby@niles.edu.eg (S.H.-E.); 2 Department of Physics, Faculty of Science, Ain-Shams University, Cairo Governorate 11566, Egypt; helghandoor@gmail.com; 3 Department of Laser Sciences and Interactions, National Institute of Laser Enhanced Sciences (NILES), Cairo University, Giza Governorate 12613, Egypt; hamed_kandel@niles.edu.eg

**Keywords:** articular cartilage, surface roughness, laser speckle, co-occurrence matrix, Haralick’s texture feature

## Abstract

The application of He-Ne laser technologies for description of articular cartilage degeneration, one of the most common diseases worldwide, is an innovative usage of these technologies used primarily in material engineering. Plain radiography and magnetic resonance imaging are insufficient to allow the early assessment of the disease. As surface roughness of articular cartilage is an important indicator of articular cartilage degeneration progress, a safe and noncontact technique based on laser speckle image to estimate the surface roughness is provided. This speckle image from the articular cartilage surface, when illuminated by laser beam, gives very important information about the physical properties of the surface. An experimental setup using a low power He-Ne laser and a high-resolution digital camera was implemented to obtain speckle images of ten bovine articular cartilage specimens prepared for different average roughness values. Texture analysis method based on gray-level co-occurrence matrix (GLCM) analyzed on the captured speckle images is used to characterize the surface roughness of the specimens depending on the computation of Haralick’s texture features. In conclusion, this promising method can accurately estimate the surface roughness of articular cartilage even for early signs of degeneration. The method is effective for estimation of average surface roughness values ranging from 0.09 µm to 2.51 µm with an accuracy of 0.03 µm.

## 1. Introduction

Articular cartilage is a glassy-like tissue that covers the ends of joint bones [1]. It supplies shock absorbent of the body and reduces the friction between the interacting bones to permit the movements of one bone against another [1,2]. Osteoarthritis is one of the most common disease of joints worldwide that describes articular cartilage degeneration and breakdown [1]. Joint injuries, excessive loading, obesity and aging can affect osteoarthritis [3]. Investigating the surface roughness of the articular cartilage is of great importance to assess the irregularity of the surface.

Many imaging tools have been used to detect the signs of cartilage degeneration. Plain radiography, the simplest and cheapest imaging tool, is insensitive to soft tissue [1,3]. As direct visualization of articular cartilage is impossible by plain radiography, it depends on the joint spacing to indirectly estimate the cartilage thickness and integrity [3,4]. Thus, plain radiography cannot detect early signs of osteoarthritis. Alternatively, Arthroscopy is used for direct visualization and evaluation of cartilage surface, however it cannot detect most cartilage injuries [5]. On the other side, arthroscopy is limited in clinics due to its high cost. Rather than direct visualization, magnetic resonance imaging tool (MRI) has the advantage of direct imaging of the articular cartilage (sensitive to soft-tissue). Therefore, it gives accurate assessment of cartilage thickness and volume, and good detection of cartilage abnormalities [1,5]. Nevertheless, MRI is very expensive, time consuming and requires rest of children [1]. As a substitute, computer tomography (CT) provides a good evaluation tool for the surface and thickness of articular cartilage [3]. However, CT has many disadvantages for some patients, such as blood pressure drop and abdominal pain. It is not recommended using it with patients suffering from kidney disease for fear of occurrence of renal failure. Besides, exposing patients to ionizing radiations is a main drawback of using CT [3].

Other measuring tools, such as atomic force microscopy (AFM), high frequency ultrasound, Laser scanning confocal microscopy, scanning white light interferometry and optical profilometry, have been used for measuring the roughness of articular cartilage surface [6]. Nevertheless, AFM is a contact method that can deform and distort the cartilage tissue [6,7] while the others have been carried out on animals and rarely on human cartilage specimens [6]. Another drawback of AFM is its very small scanning area (~100 µm^2^) for measuring the cartilage roughness [8].

Laser speckle has been widely used in material engineering to estimate the surface roughness of different materials for many applications. Laser speckle is simple, safe and noncontact method that can carry a lot of information about the surface deformation. It is based on the phenomenon that, when a coherent light beam illuminates a rough surface, it scatters randomly in all directions resulting in an illuminated image with bright and dark spots. This image, called laser speckle image, results from the random interference of the scattering rays. The bright spots correspond to constructive interference while the dark spots result from destructive interference [9,10]. Laser speckle image can be observed either in free space (objective speckle) or on the image plane of an imaging lens (subjective speckle) [9]. Accordingly, in this work, a robust technique has been proposed to evaluate the surface roughness of the articular cartilage to increase the accuracy of early detection of articular cartilage deformation. When the articular cartilage surface is illuminated by a laser beam, it can be reflected, scattered or absorbed depending on the beam wavelength and the surface structure [11]. As demonstrated in [12], articular cartilage showed very weak absorption properties in the wavelength range ~400–850 nm with the domination of the scattering properties. Therefore, red He-Ne laser operating at 632 nm was used in this study. In addition, red He-Ne laser is widely used in material engineering over diode laser for its highly spatial and temporal coherence [9].

Many different statistical parameters can be extracted from the laser speckle image that can be used to evaluate the surface roughness or to differentiate between different surfaces. Shulev et al. [13] used the average speckle size to measure the roughness of zirconia materials, used for dental crowns. They computed the average speckle size from the normalized autocorrelation method of the speckle image. Besides the normalized autocorrelation method, shifting the image in x-direction, y-direction and diagonal direction were used to evaluate the roughness of metal surfaces [14,15]. The laser speckle contrast method using first-order statistics was applied to relate the speckle contrast to the surface roughness [16,17]. However, it can evaluate surface roughness values up to 0.2 µm [17]. Measurement of metal surface roughness using statistics of binary speckle images showed that there is a strong relation between the surface roughness of the inspected surfaces and the parameters extracted from the binary image [18,19,20]. Texture analysis of laser speckle images using the co-occurrence matrix of the speckle images was applied to measure the roughness of paper and specimens [21,22].

The proposed method is based on computing some texture features from an implemented gray level co-occurrence matrix (GLCM) of the laser speckle image that describe the cartilage surface. GLCM depends on second order statistics, as it deals with the properties of two neighbor pixels, contrary to the first order statistics that deal with the properties of the individual pixels, computing average and standard deviations.

## 2. Results

### 2.1. Laser Speckle Images

**Figure 1:**
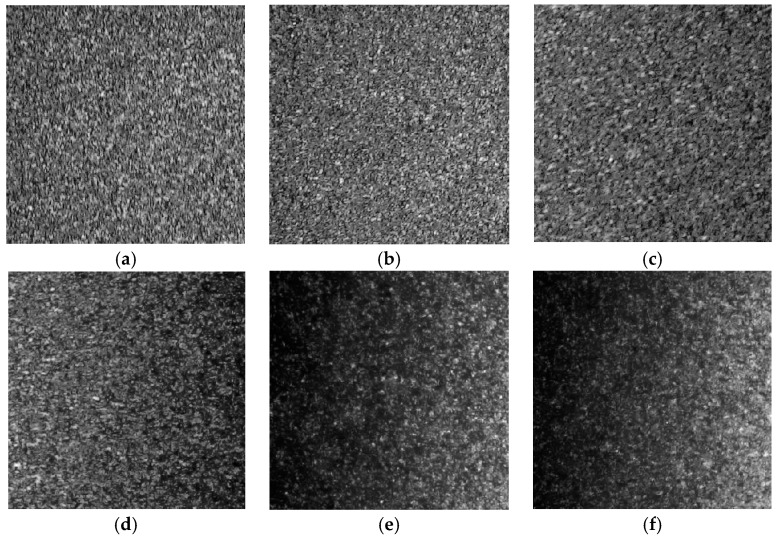
Laser speckle images for six articular cartilages with different average surface roughness values of: (**a**) R_a_ = 0.09 µm; (**b**) R_a_ = 0.33 µm; (**c**) R_a_ = 0.43 µm; (**d**) R_a_ = 0.77 µm; (**e**) R_a_ = 2.08 µm; and (**f**) R_a_ = 2.51 µm.

The laser speckle images presented in Figure 1 were captured using the experimental setup shown in Section 4.1 The presented images were obtained from the scattered rays of six articular cartilage specimens with different average surface roughness (R_a_) values of 0.09, 0.33, 0.43, 0.77, 2.08 and 2.51 µm (see Section 4.2). They are 8-bit gray-level images having a dimension of 400 × 400 pixels that consist of bright spots (speckle grains) and dark spots.

**Figure 2:**
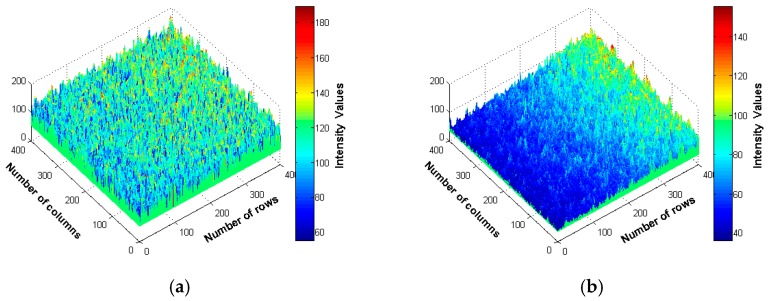
Three-dimensional intensity distribution of speckle images: (**a**) R_a_ = 0.33 µm; and (**b**) R_a_ = 2.51 µm.

**Figure 3:**
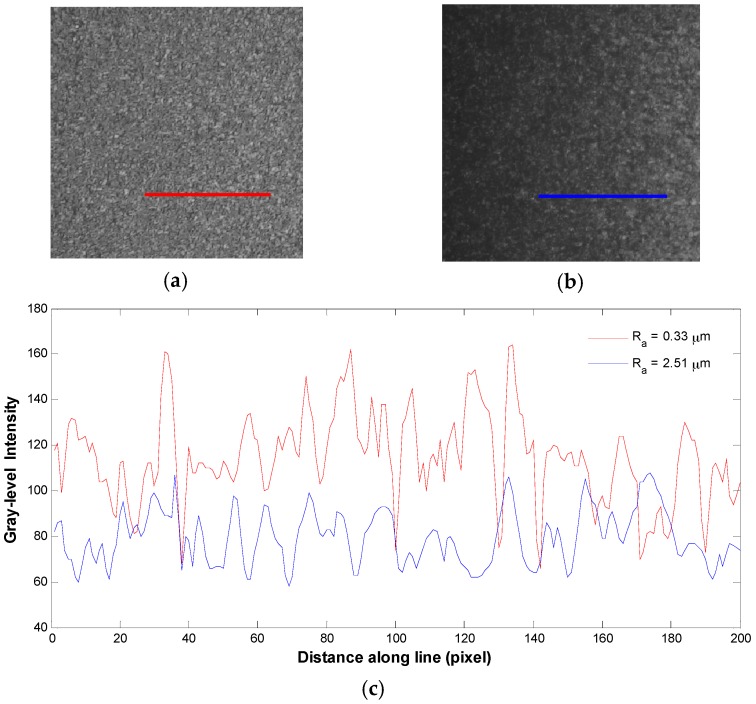
Line plot of gray-level intensities: (**a**) speckle image of R_a_ = 0.33 µm; (**b**) speckle image of R_a_ = 2.51 µm; and (**c**) gray-level intensities along the lines passing over the two speckle images.

For better view of the speckle image texture, three-dimensional plots of the gray-level intensity distribution of the speckle images for smooth and rough specimens are provided in Figure 2. Additionally, gray-level intensities along a line in the two speckle images are extracted and plotted in Figure 3 for comparison.

### 2.2. GLCM Analysis

The GLCM of the obtained speckle images is constructed depending on the neighbor pixels distance (d) and the neighbor pixels direction (θ) (more details are presented in Section 4.3).

#### 2.2.1. Effect of Neighbor Pixels Direction

**Figure 4:**
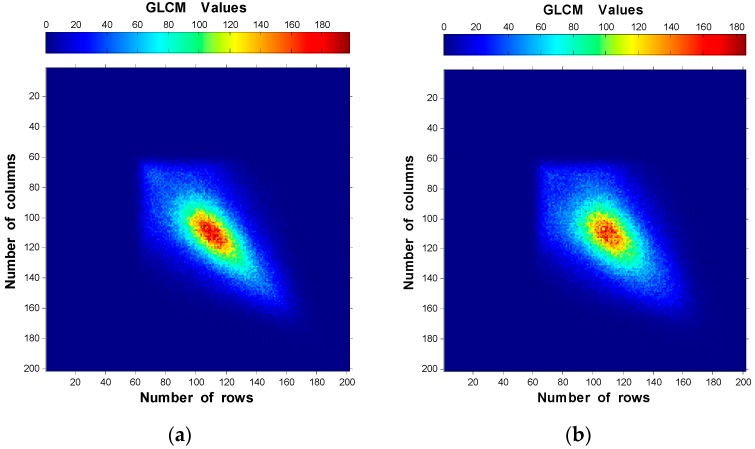

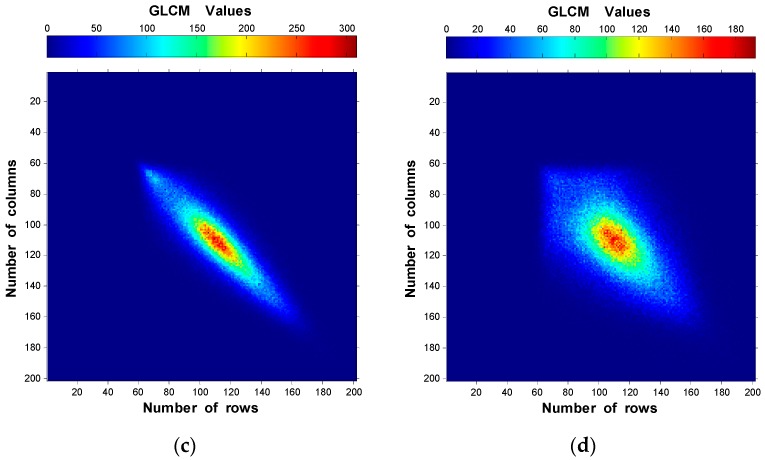
GLCM plots of R_a_ = 0.09 µm using d = 1 pixel along the four directions: (**a**) 0°; (**b**) 45°; (**c**) 90°; and (**d**) 135°.

The GLCM of the specimen of R_a_ = 0.09 µm along the four neighbor pixels directions using d = 1 pixel were modeled and plotted in Figure 4. It is obvious from the obtained results that the elements distribution of the GLCM along 90° direction shows lower spreading away from the main matrix diagonal than other directions with the highest GLCM value (accumulation of elements).

#### 2.2.2. Effect of Neighbor Pixels Distance

**Figure 5:**
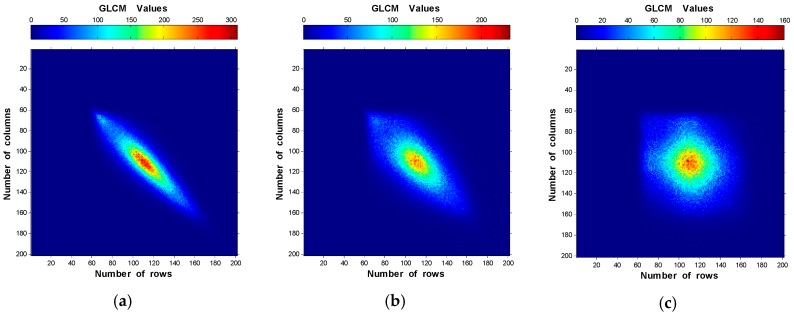

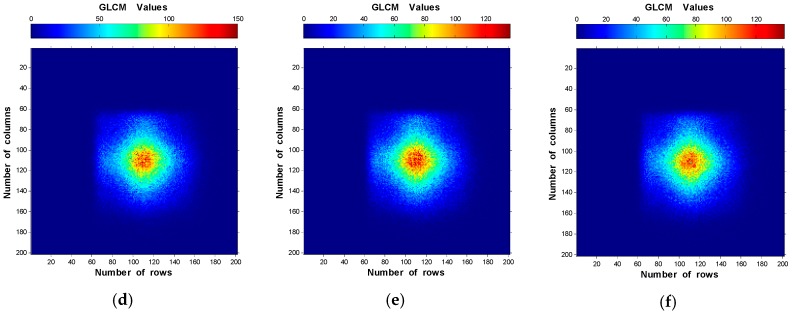
GLCM plots of R_a_ = 0.09 µm along 90° direction using neighbor pixels distances of: (**a**) 1 pixel; (**b**) 2 pixels; (**c**) 5 pixels; (**d**) 10 pixels; (**e**) 20 pixels; and (**f**) 30 pixels.

To study the effect of the neighbor pixels distance, some GLCM of one of the specimens were calculated and plotted in Figure 5. The results present 2-D plots of the GLCM along 90° direction using different neighbor pixels separation. The plots indicate that increasing the neighbor pixels distance increases the elements distribution width around the main diagonal of the GLCM until a certain limit (using d ~ 10–30 pixels) where it becomes nearly constant with nearby maximum accumulation values of the matrix. Very similar results are shown in Figure 5e,f.

#### 2.2.3. Assessment of GLCM

**Figure 6:**
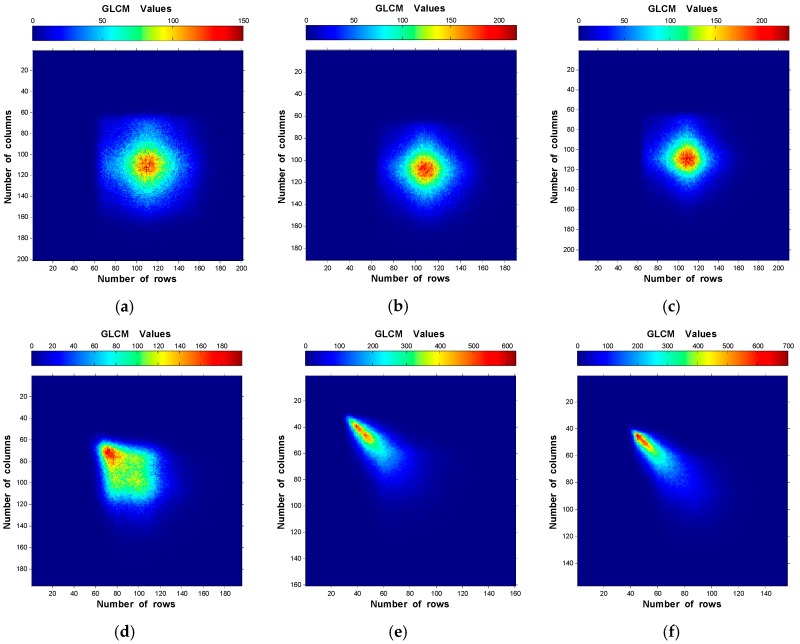
Two-dimensional plots of GLCM using d = 10 pixels along 90° direction of the six speckle images of: (**a**) R_a_ = 0.09 µm; (**b**) R_a_ = 0.33 µm; (**c**) R_a_ = 0.43 µm; (**d**) R_a_ = 0.77 µm; (**e**) R_a_ = 2.08 µm; and (**f**) R_a_ = 2.51 µm.

From the results shown in Figure 4 and Figure 5, the GLCM was analyzed along 90° direction using d = 10 pixels for the speckle images of the six specimens. The obtained results are provided in Figure 6. One can observe that the elements of the GLCM for rough surface distribute closer to the main diagonal of the matrix, showing higher accumulation than for smooth surface. Besides, The GLCM elements move towards the upper side of the main matrix diagonal for higher R_a_ values.

### 2.3. GLCM’s Texture Features Analysis

**Figure 7:**
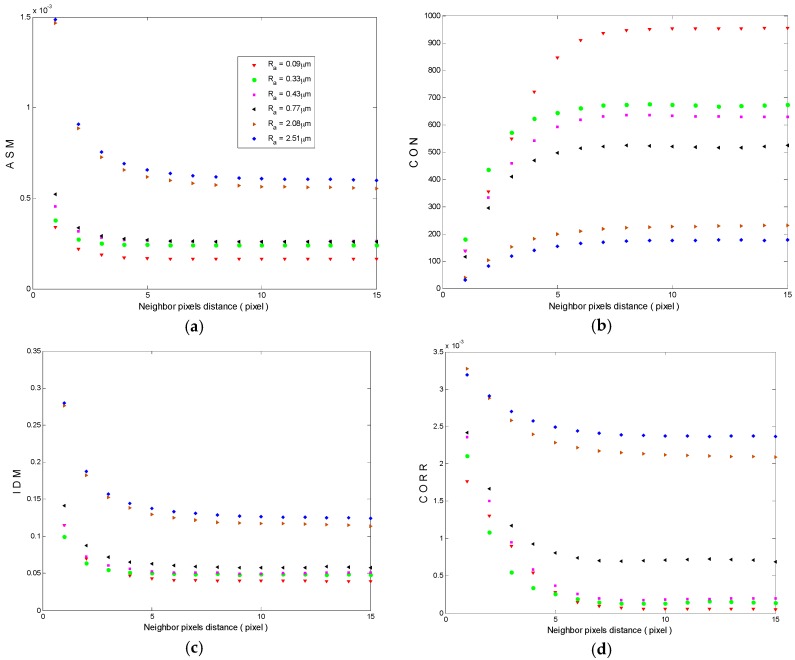
Haralick’s texture features versus the neighbor pixels distance: (**a**) angular second momentum; (**b**) contrast; (**c**) inverse different moment; and (**d**) correlation.

**Figure 8:**
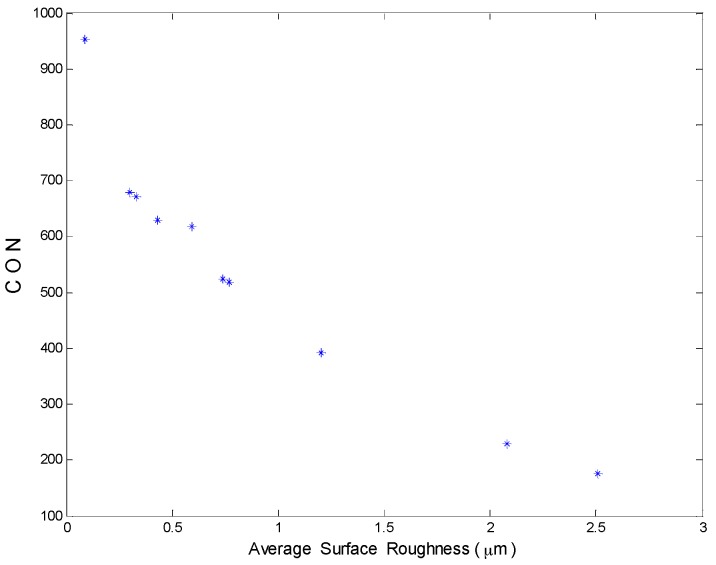
Contrast feature with respect to average surface roughness.

GLCM was calculated for each specimen along 90° direction using neighbor pixels separation in the range of 1–15 pixels. For each obtained GLCM, the angular second moment (ASM), contrast (CON), inverse difference moment (IDM) and correlation (CORR) features are computed (Section 4.4). The results of these features for the six specimens with respect to the neighbor pixels distance is presented in Figure 7. In this figure, it is observed that the feature curves versus neighbor pixels distance gives nearly the same results after d = 8 pixels in all cases. Besides, it is clearly observed that all features have a good relationship with the surface roughness. The curves in Figure 7a,c,d show that ASM, IDM and CORR are not sensitive enough for smooth surfaces where, overlapped and coincided results were obtained for nearby surface roughness. On the other hand, CON (Figure 7b) is very sensitive for rough and smooth surfaces. These observations with the above results shown in Figure 5 dictated to us the necessity to simplify data manipulation. Consequentially, for each specimen, only six GLCM using d in the range of 10–15 pixels were calculated and then CON was computed from these six matrices and averaged. Figure 8 shows the relationship between the averages of the calculated CON and the average surface roughness of 10 articular cartilage specimens with different average roughness values.

## 3. Discussion

It can be seen in Figure 2 and Figure 3 that the intensities of the speckle grains become weaker with increasing surface roughness because the increasing scattered rays yield fewer photons to the camera. Moreover, the average speckle grain size increases as the surface roughness increases and the rough surface contains more dark areas than smooth surface. Hence, these results imply that speckle image holds important information about the surface roughness that can be extracted by texture analysis using gray-level co-occurrence matrix of the speckle image.

Figure 4, Figure 5 and Figure 6 show the elements distributions of the GLCM. It is obvious that the elements distribute around the main matrix diagonal due to the speckle texture which mainly contains bright and dark spots (few gray-levels). Furthermore, the elements move towards the main matrix diagonal as the roughness increases (Figure 6) due to the domination of lower intensities. This can be expressed as: the rough surface has large speckle grains that tend to increase the number of similar neighbor pixels (homogeneity increase). Another explanation of this is that there is less gray-level transition between the neighbor pixels for rough surface than smooth one. Therefore, fewer entries with large values to the GLCM near the diagonal are obtained. On the other hand, high variations between neighbor pixels (very smooth surface) give large entries of small values to the GLCM away from the main diagonals. Then, as the speckle grain size decrease, the elements accumulate close to the main diagonal.

The results of the angular second momentum feature provided in Figure 7a for the six specimens show that, as the surface roughness increases the ASM (the sum of squares of the GLCM elements (Equation (4)) increases due to the domination of few numbers with large magnitude). However, ASM has a good relationship with average surface roughness; it is not sensitive enough to close roughness values, especially for smooth surfaces because ASM values are in the range of 0–1. As shown in Figure 7a, nearby ASM values for the specimens with R_a_ values of 0.33, 0.43 and 0.77 µm are obtained.

The result in Figure 7b shows that the contrast feature decreases as the surface roughness increases. As demonstrated before, the higher is surface roughness, the greater is the speckle grain size and therefore the lower is weight factor (Equation (5)), which is dominant. Then, lower contrast feature is predicted for higher roughness. 

Because the homogeneity of pixels in speckle image increases when the speckle grain size increases, the inverse difference moment and correlation features values increase. Realistic results are obtained for IDM and CORR in Figure 7c,d respectively. 

From the results shown in Figure 7, it has been found that all features have a good relationship with surface roughness where, ASM, IDM and CORR increase by increasing surface roughness, while CON decreases by increasing surface roughness. Hence, all of them can be used for the estimation of surface roughness of articular cartilage.

Nevertheless, IDM add a weight factor to the elements with respect to their places (Equation (6)), and less sensitivity for closest values of surface roughness are obtained, as obviously shown in Figure 7c. The reason is that its weight factor is the inverse to the contrast weight factor, which results in very law values (less than 1). Besides, the very small values of CORR make it less sensitive to nearby roughness values.

However, CON is the most suitable feature for surface roughness estimation, as it is very sensitive to all roughness values as obvious in Figure 8, where it can distinguish the nearby average roughness values of 0.30 and 0.33, and 0.74 and 0.74 µm. The assumed reason is that CON favors the GLCM elements with a weight factor according to their places from the main diagonal that result in its higher values.

## 4. Materials and Methods

### 4.1. Experimental Setup

**Figure 9:**
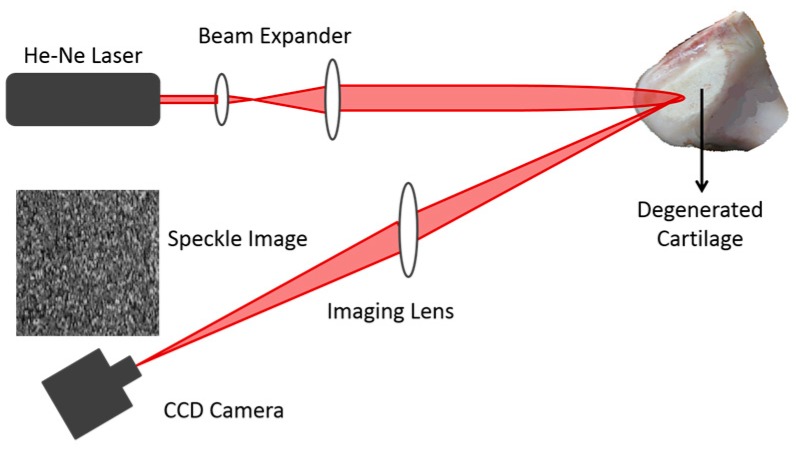
The optical setup used for recording speckle images.

The optical setup used for recording of laser speckle images is shown in Figure 9. To build the setup, a collimated He-Ne laser beam of constant power 1 mW operating at a wavelength of 632 nm illuminates a cross section area (~36 mm^2^) of the cartilage specimen after passing through a beam expander. The scattered light from the specimen is then collected with a digital camera located at the image plane of an imaging lens to obtain the speckle image which is formed due to the random interference that takes place at the image plane (subjective speckles). The average size of the speckle grains (bright spots) is related to the laser wavelength λ and the imaging lens using Equation (1) as follows [15]:

(1) $$\delta \;=\;1.22\;\lambda \;(1\;+\;M)\;\frac{f}{D}$$

where f is the focal length of the imaging lens, D is the imaging lens diameter and M is the magnification. Hence, the recorded speckle images for the different specimens have been recorded under the same experimental conditions, i.e., using the same parameters of λ, f, M and D. Moreover, the selected cross section area, which has been used for recording the speckle images was kept constant for all specimens at a fixed room temperature. In addition, the angle between the incident laser beam and the normal direction is fixed at 15° for all specimens.

### 4.2. Specimen Collection and Preparation

Ten articular cartilage specimens were collected from two bovine knee joints at the butcher’s shop and prepared using abrasive machine for different roughness values. Their average surface roughness, R_a_, values were measured using a stylus device. The specimens were transported to the lab in ice box and stored in the freezer until used in the experiment. Before imaging process, they were left in room temperature for 6 hours and all the specimens were disposed immediately after image collection. The prepared specimens were divide into two main groups. The first group contains six specimens of smooth surfaces (with average surface roughness of 0.09, 0.33, 0.43 and 0.77 µm) and rough surfaces (with average surface roughness of 2.08 and 2.51 µm) that was intended to be used for studying the proposed system. The second group (contains four specimens with R_a_ values of 0.30, 0.59, 0.74 and 1.2 µm) was used to enrich data analysis.

### 4.3. Extraction of Gray-Level Co-Occurrence Matrix

Texture analysis describes the spatial variation of an image based on extracting some statistical parameters from the image. Haralick et al. [23], innovated a method that can extract 14 texture features based on two steps. The first step was to implement a gray-Level co-occurrence matrix and the second one was to compute texture features from this matrix. The GLCM deals with second-order statistics where it is based on how often two-pixel values separated by a certain distance d and lie along a certain direction θ are related.

Assume that the speckle image F has size M × M pixels and gray-level values L = {0,1,2,….,G−1} with horizontal and vertical dimensions of M = {1,2,…,m} and N = {1,2,….,n}, respectively. Then, each element of a G × G gray-level co-occurrence matrix (CM) is the number of times that any two pixels (x,y) and (x + d,y + d) with gray-level values of F(x,y) = i and F(x + d,y + d) = j, respectively, separated by a distance d along a direction of θ = {0°,45°,90° or 135°} are repeated [23]. Hence, for simplicity these elements can be defined by the following equation:

(2) $${\mathrm{CM}(i,j)}_{d,\theta }=\;\overset{m}{\underset{x\;=\;1}{\sum }}\overset{n}{\underset{y\;=\;1}{\sum }}\{\begin{array}{c} 1,\;\mathrm{if}\;F\left(x,y\right)\;=\;i\;\mathrm{and}\;F\left(x\;+\;d,y\;+\;d\right)\;=\;j\\ 0,\;\mathrm{otherwise} \end{array}$$

**Figure 10:**
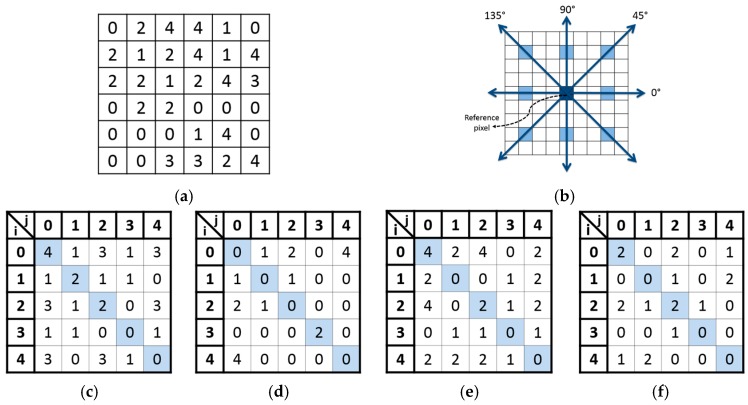
GLCM calculation for the sample image using d = 3 along the four directions: (**a**) sample image; (**b**) reference pixel and its eight neighbors at d = 3; (**c**) GLMC along 0° direction; (**d**) GLMC along 45° direction; (**e**) GLMC along 90° direction; and (**f**) GLMC along 135° direction.

Figure 10 shows the calculated GLCM along the four directions of θ for a sample image of size 6 × 6 contains five gray-level values (Figure 10a). It must be mentioned that, the GLCM is symmetric since the calculation is maintained in the forward and backward directions as obvious in Figure 10b. The highlighted cells of the four GLCM specify the main diagonal of the matrix (Figure 10c–f).

The elements of GLCM are then normalized to give the probability of GLCM as given by Equation (3) [23]:

(3) $$P\left(i,j\right)\;=\;\frac{\mathrm{CM}\left(i,j\right)}{\sum_{i\;=\;0}^{G-1}\sum_{j\;=\;0}^{G-1}\mathrm{CM}\left(i,j\right)}$$

where i and j are the horizontal and vertical spatial dimensions of CM, G is the maximum gray-level value in the image and P is the normalized GLCM. Thus, the sum of the P elements is equal to one.

### 4.4. Texture Features Extraction from GLCM

The four GLCM contain information about the distribution and relationships between the neighbor pixels of the speckle image. Therefore, the main Haralick’s texture features must be computed; the angular second moment, contrast, inverse difference moment and correlation.

The angular second moment feature is also known as uniformity or energy. It measures the uniformity of an image. ASM is defined as [23]:

(4) $$\mathrm{ASM}\;=\;\overset{G-1}{\underset{i\;=\;0}{\sum }}\overset{G-1}{\underset{j\;=\;0}{\sum }}P^{2}\left(i,j\right)$$

It can be seen from this equation that the ASM value is proportional to the square of the GLCM elements. When there are many gray-level transitions between the speckle image pixels, there will be large numbers of small entries to the GLCM and the ASM value will be small. On the other hand, if the image is slowly varying, fewer entries to the GLCM with large magnitudes will dominate.

The contrast feature is a measure of the gray-level intensity variations between the pixels to show the image intensity contrast. It is also known as inertia and is given by [23]:

(5) $$\mathrm{CON}\;=\;\overset{G-1}{\underset{n\;=\;0}{\sum }}n^{2}\{\overset{G-1}{\underset{i\;=\;0}{\sum }}\overset{G-1}{\underset{j\;=\;0}{\sum }}P\left(i,j\right)\}$$

where n = |i−j| represents a weight factor. The same neighbor pixels in the speckle image will fill the GLCM main diagonal and the diagonal is modified by a weight factor n = 0. If the neighbor pixels i and j differ by 1, then n = 1 and there is a small increase in the contrast feature. Hence, as the difference between the two neighbor pixels increases, the weight factor increases and consequently, the contrast feature increases [24].

The inverse difference moment feature which is also named homogeneity, measures how the elements of the GLCM close to the GLCM diagonal. Thus, as observed in Equation (6) [23], its weight factor is the inverse of the contrast weight factor. Therefore, the homogeneity feature increases with increasing the very similar neighbor pixels.

(6) $$\mathrm{IDM}\;=\;\overset{G-1}{\underset{i\;=\;0}{\sum }}\overset{G-1}{\underset{j\;=\;0}{\sum }}\frac{1}{{1\;+\;(i\;-\;j)}^{2}}\;P\left(i,j\right)$$

The correlation feature describes the correlations between the pixels in the rows and columns of the GLCM as expressed by Equation (7) [23,24].

(7) $$\mathrm{CORR}\;=\;\overset{G-1}{\underset{i\;=\;0}{\sum }}\overset{G-1}{\underset{j\;=\;0}{\sum }}P(i,j)\frac{{(i\;-\;\mu }_{x}{)(j\;-\;\mu }_{y})}{\sigma_{x}\sigma_{y}}$$

the correlation feature is based on the means of rows ($\mu_{x}$) and columns ($\mu_{y}$), and the standard deviations of rows ($\sigma_{x})$ and columns ($\sigma_{y}$). As the GLCM is symmetrical, $\mu_{x}{\;=\;\mu }_{y}$ and $\sigma_{x\;}{=\;\sigma }_{y}$. They are calculated using the following equations:

(8) $$\mu_{x}\;=\;\overset{G-1}{\underset{i\;=\;0}{\sum }}\overset{G-1}{\underset{j\;=\;0}{\sum }}\mathrm{iP}(i,j)$$

(9) $$\mu_{y}\;=\;\overset{G-1}{\underset{i\;=\;0}{\sum }}\overset{G-1}{\underset{j\;=\;0}{\sum }}\mathrm{jP}(i,j)$$

(10) $$\sigma_{x}\;=\;\overset{G-1}{\underset{i\;=\;0}{\sum }}\overset{G-1}{\underset{j\;=\;0}{\sum }}{{(i\;-\;\mu }_{x})}^{2}P(i,j)$$

(11) $$\sigma_{y}\;=\;\overset{G-1}{\underset{i\;=\;0}{\sum }}\overset{G-1}{\underset{j\;=\;0}{\sum }}{{(j\;-\;\mu }_{y})}^{2}P(i,j)$$

## 5. Conclusions

Investigations into a method that can measure the surface roughness of articular cartilage to overcome the problems of other imaging modalities (as demonstrated in Section 1) have been proposed and described in detail. The method was based on second order texture analysis of laser speckle image. It was intended to use laser speckle imaging technique as it is a simple, inexpensive and safe method that has been implemented with a very low power laser. Besides, it is a noncontact method that will not distort the articular cartilage surface tissue during the imaging process using the optical setup.

The main Haralick’s texture features have been calculated from the implemented gray-level co-occurrence matrix based on the obtained laser speckle images for 10 articular cartilage specimens. Contrast feature showed a good illustration of the surface roughness for all the specimens and therefore minute roughness changes can be easily distinguished with high measuring accuracy up to 0.03 µm. Although angular second momentum, inverse difference moment and correlation features show good results, they are not sensitive enough to smooth surfaces for nearby roughness. Hence, the contrast feature has turned out to be the best feature to estimate surface roughness.

Finally, this study highlighted the accuracy of laser speckle imaging for the estimation of early signs of articular cartilage degeneration.

In future work, it is planned to complete the study to benefit from the combination of the different Haralick’s texture features together for the best accuracy in roughness estimation.
